# The Wound Healing Effect of* Callicarpa nudiflora* in Scalded Rats

**DOI:** 10.1155/2019/1860680

**Published:** 2019-05-02

**Authors:** Xu-guang Zhang, Xiu-min Li, Xin-xin Zhou, Yong Wang, Wei-yong Lai, Ying Liu, Yu-chao Luo, Jun-qing Zhang

**Affiliations:** ^1^Department of Pharmacy, Hainan Medical University, No. 3 Xueyuan Road, Longhua District, Haikou 571199, China; ^2^Hainan Provincial Key Lab of R&D on Tropic Herbs, Hainan Medical University, No. 3 Xueyuan Road, Longhua District, Haikou 571199, China; ^3^Department of Pharmacy, Harbin University of Commerce, 138 Tongda Street, Daoli District, Harbin 150076, China

## Abstract

*Callicarpa nudiflora* has been widely used in Li nationality medicine and treated burns and scalds in China. Our objective was to preliminarily elucidate healing effect and action mechanism of* Callicarpa nudiflora* water extract (CNE) on the scald wounds using an experimental rat mode. The second-degree scald wounds were induced by hot water on dorsal surface of Sprague-Dawley (SD) rats, and then they were randomly divided into 5 groups as follows: control (CON), Vaseline, Silver sulfadiazine (SSD), and Vaseline supplemented with 10% and 20% CNE groups. These ointments were employed locally once daily for 21 days. The macroscopic analysis showed CNE significantly accelerated the wound healing process and lowered the wound areas on days 15, 18, and 21 especially in 20% CNE group compared to CON group. Histopathological evaluation showed the mildly hypertrophic epidermis and the intact dermis in the 20% CNE-treated group were obviously distinguished from CON group on day 21. The CNE-treated groups had no obvious effect for TNF-*α* and IL-10 expressions on the second day and 14th day, while TGF-*β*1 expression level was decreased on the 21th day and VEGF level was increased on the 7th day in the 20% CNE group. Furthermore, the expression level of Samd3 was strongly inhibited in 20% CNE group. These findings suggested that the CNE can enhance the wound healing and skin repair in deep second-degree scald rats and thus support its traditional use.

## 1. Introduction

Deformation of appearance and skin dysfunction caused by serious pathological scar largely hyperplastic contracture resulting from scald is a huge difficulty for clinical treatment [1]. Chronic, nonhealing wound and their treatment will also cause considerable socioeconomic losses in developing countries [2, 3]. Vaseline as a classic and common ointment base is often used for burns, wounds and other skin lesions. And silver sulfadiazine cream (SSD) is the most widespread topical treatment for burns, toxic epidermal necrolysis wounds, bullous disorders, and ulcers [4]. However, there are also some potential side effects simultaneously including leukopenia when applied to large burn surfaces, hypersensitivities, delays in the wound healing processes, and serious cytotoxic activity in the host cells [5–7]. Luckily, several traditional Chinese medicines of plant origin have been employed to clinical research on the wound treatments without obvious side effects [8, 9]. The use of botanical drug is an effective method in improving healing of burn and scald wounds and reducing fiscal burden [10]. Therefore, the stable, eutherapeutic, and cheaper drugs are needed in the wound management.

In China, numerous* Callicarpa* species herbs are served as Chinese folk medicines, Li nationality medicines, which treat various indications.* Callicarpa nudiflora* Hook. et Arn. (*C. nudiflora*), one of the most widely used as Li nationality medicine in China, belongs to genus* Callicarpa*, family Verbenaceae, distributing in subtropical or tropical areas such as the southern of China. The stems and leaves of* C. nudiflora* possess powerful medicinal values due to its antibacterial, hemostatic, and anti-inflammatory properties, wherefore it is mainly employed to some indication remedies, such as bleeding, inflammation, and infection [11–13]. Moreover,* C. nudiflora* can be also used externally for burns and scalds [14], which has been ignoring for a long time. According to medicine theory, wound healing involves a sequence of events including inflammation, angiogenesis, cell migration, matrix synthesis, and reepithelialization [15]. And researchers have demonstrated that the presence of active chemical constituents in* C. nudiflora* displayed in vitro and in vivo anti-inflammation, wound healing, and hemostasis [16–18] thereby playing a role in wound healing effects of burns and scalds. But there is almost no scientific report about the research on its action mechanism of wound healing. And how to exert wound treatment effects of* C. nudiflora* still remains unknown.

Hence, our current research objective is to preliminarily illustrate scald wound healing efficacies and action mechanisms of the water extract of* C. nudiflora* (CNE) using an experimental SD rat model.

## 2. Materials and Methods

### 2.1. Preparation of CNE

The collected stems and leaves of* Callicarpa nudiflora* were from Haikou city of China and identified by Professor Niankai Zeng of Hainan Medical University.* Callicarpa nudiflora* used in this study was deposited in Hainan Medical University, Haikou, China. 1.0 Kg dried stems and leaves (1:1 w/w) were powdered and extracted two times for 2h with water through heating reflux. Then aqueous extract was filtered and concentrated using the rotary vacuum evaporator. The yield of crude extract was 19.99%.

### 2.2. Reagents

White Vaseline and 1% SSD were obtained from Zhiyuan Chemical Reagent Co. Ltd. (Tianjin, China) and Kunming Shenghuo Pharmaceutical Co. Ltd. (Kunming, Yunnan, Korea), respectively. Rat enzyme-linked immunosorbent assay kits of interleukin-10 (IL-10), tumor necrosis factor-*α* (TNF-*α*), transforming growth factor-*β*1 (TGF-*β*1), and vascular endothelial growth factor (VEGF) were purchased from Biocalvin Co. Ltd. (Suzhou, Jiangsu, China). *β*-actin polyclonal antibody and rabbit anti-Smad3 were obtained from Abcam Co. Ltd. (Cambridge, UK).

### 2.3. Animals

SD adult male rats were used for the experiment and the weight of each rat was between 180g and 200g. They were housed individually per cages and allowed ad libitum access to tap water and the commercial balanced rat diet.

### 2.4. Induction and Treatment of Scald Wound

24h before creating scald wound, rat dorsal hairs were shaved and untreated hairs were removal with 8% sodium sulfide solution, and then 0.1% benzalkonium bromide was used to sterilize the dorsal area. The scald wound rat model was built with a method described by previous study with some modifications [19]. The second-degree scald wound was induced by hot water (90°C for 15 s) on the dorsal surface and the size of the wound was approximately 7.5cm^2^ (3cm × 2.5cm). The scald rats were then randomly assigned to 5 groups (Low-dose, High-dose, SSD, Vaseline, and Control (CON) groups, n = 8, respectively). The scald rats in Low-dose and High-dose groups were applied with the Vaseline ointments containing 10 and 20% (w/w) CNE. The scald rats in the SSD and Vaseline groups were treated with the 1% (w/w) SSD cream and Vaseline, respectively. And the CON group was not treated after scald. 24 h later, 0.3 ml of prepared substances in all groups were applied to scald wounds once a day for three weeks.

### 2.5. Macroscopic Analysis of Scald Wound

Scald areas were evaluated everyday by standards as follows: the colors of wound beds, secretions, firmness, and swelling on the surfaces of scald wounds. On days 1, 5, 8, 12, 15, 18, and 21 following the scald injury, optical photographs were taken and analyzed using ImageJ to calculate areas of wounds. The following formula was used for calculating the wound contraction rates: Contracture rate = Wound sizes in the specific day / Wound sizes in the original state × 100% [20, 21].

### 2.6. Collection and Processing of Biological Samples

Whole blood of scald rats was collected from the orbital sinus on days 2, 7, 14, and 21. The rat serum was gained by centrifuging the collected blood (3,000 rpm, 15 min) which was stored at −80°C before the tests. The scald areas and the surrounding wound margin tissues of 21 days were excised to the muscle fascia levels and stored at −80°C until analysis [20, 21].

### 2.7. Histopathological Study with Hematoxylin-Eosin (HE) Staining

Rats were sacrificed at day 21 after scald using ether, and skin samples were taken for histopathological study. The skin samples were fixed in 10 % formalin solution. After fixation, the tissues were washed in running tap water, dehydrated in ascending grades of ethyl alcohol, and cleared in xylene. Paraffin embedded tissue sections of 6-*μ*m thickness were cut using a microtome and mounted on glass slides. Histological sections were stained with HE for histological examination. Digital photomicrographs were captured at representative locations using a digital camera attached to a microscope. Tissue samples were evaluated for the extent of dermal bleedings, epidermal exfoliation and scabbing, inflammatory cell responses, and proliferations of fibrous tissues.

### 2.8. Enzyme-Linked Immunosorbent (ELISA) Assay

Rats' serum TNF-*α*, IL-10, VEGF, and TGF-*β*1 were quantitatively assayed using respective ELISA kits. The experiments were conducted on the basis of manufacturer's instructions of ELISA kits. Each cytokine in all samples was tested three times, and the mean of the result data was expressed as pg/mL or ng/mL [19].

### 2.9. Western Blot Analysis

Scald tissues of 21 days were diced into 1 mm pieces with a pair of ophthalmic scissors in a mortar on ice ensuring that the scissors and grinders were frozen to keep the tissue closed to the temperature of ice throughout the procedures. Then add the diced tissue to ice-cold RIPA buffer (including 100*μ*g/ml PMSF). Transfer the tissue preparation to an ice-cold tissue homogenizer and homogenize on ice for 5 × 20 seconds at 80% power. The extracts were cleared by centrifugation at 12,000RMP at 4°C for 5 min after 30 min incubation on ice, whereafter the obtained supernatant was collected and stored at -80°C before analysis. According to the manufacturer's instructions, DC protein assay kit was used to measure the total protein concentration of the supernatant from Bio-Rad and a spectrophotometer. Equal amounts of protein (45 *μ*g) were isolated by SDS-PAGE and electroblotted to polyvinylidene difluoride membrane. Immunoblots were blocked for 2 h by the mixture of tris-buffered saline and Tween 20 adding 5% skim milk, and then they were incubated with primary antibodies at 4°C the night before (rabbit anti-Smad3 and rabbit anti-*β*-actin antibodies). Membranes were washed twice using TTBS before incubating with secondary antibodies for 1.5h and Tween 20 before developing by enhanced chemiluminescence for 1-2 min, respectively. Images were scanned with Image Lab Software using ChemiDoc XRS+ System. Optical density values were analyzed using ImageJ. Smad3 protein levels were defined as the relative values of the *β*-actin protein.

### 2.10. Statistical Analysis

The values were expressed as means ± S.E.M (standard error) for more than three independent experiments for each sample. One-way analysis of variance was used for making statistical comparisons. When P values were < 0.05 the data differences were deemed to be statistically significant.

## 3. Results and Discussion

### 3.1. Gross Examination

**Figure 1:**
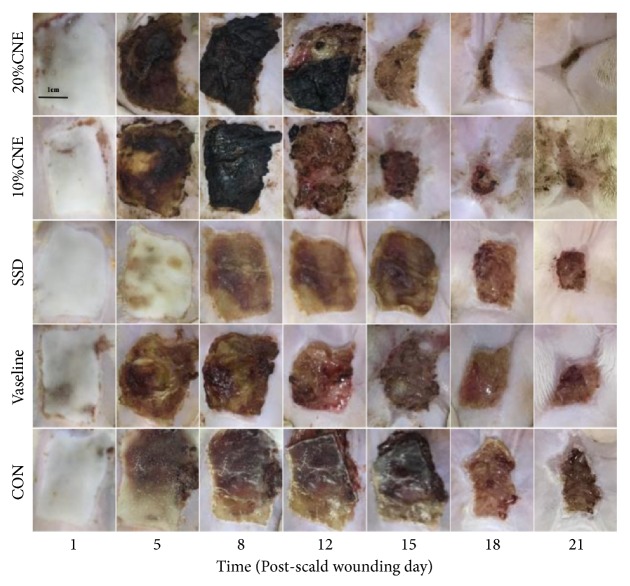
Gross appearance of scald wound. CNE, the water extract group of* Callicarpa nudiflora*; SSD, silver sulfadiazine group; CON, control group.

Scald wounds in the SSD group showed thick, dry, and yellowish-brown scabs that were intact from day 2 to day 14. The Vaseline and CNE treated group showed thick, moist, soft scabs with bleeding and exudates. However, the scabs in the CON group exhibited to be dry, thick, darken-brown. There were no measurable differences among all groups. It is well to be reminded that the colors of wounds in CNE-treated groups were dark black by the reason of the color of water extract of* Callicarpa nudiflora* itself. After day 14, the bleeding did not exist in all groups but CON group. In addition, the scabs in CNE-treated groups became much thinner than others. By day 19, the significant improvement was observed in wound closure in CNE-treated groups compared to others, especially in 20% CNE-treated group. On the 21th day, the best result was obtained in the CNE-treated groups, in which the wounds had almost healed up and the skin surfaces were smooth with the skin colors closely to be normal. The scabs in Vaseline and SSD treated groups narrowed down a lot, but still clearly existed. The skin surfaces were rough and harder than normal skin. The CON group still showed dry and dark brown scabs (Figure 1).

### 3.2. Measurement of Scald Wound Size

**Figure 2:**
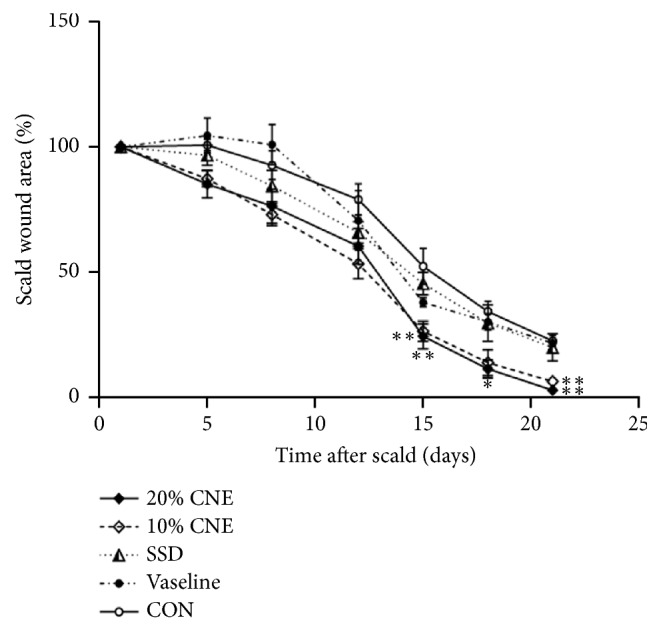
Changes in scald wound sizes. The following formula was used for calculating the wound contraction rates: Contracture rate = Wound sizes in the specific day / Wound sizes in the original state × 100%. CNE: the water extract of* Callicarpa nudiflora*; SSD: silver sulfadiazine; CON: control; values were expressed as means ± SEM. And *∗p* < 0.05 and *∗∗p *< 0.01 versus control.

The wound contraction ratio was not significant difference among the groups in the first 12 days. However, the percentage wound areas in CNE-treated groups were significantly lower than that of CON group (*p *< 0.01) in the 15th and 21st day. Furthermore, results demonstrated that the best healing effect was in the 20% CNE-treated group (Figure 2).

### 3.3. Histopathological Examination

**Figure 3:**
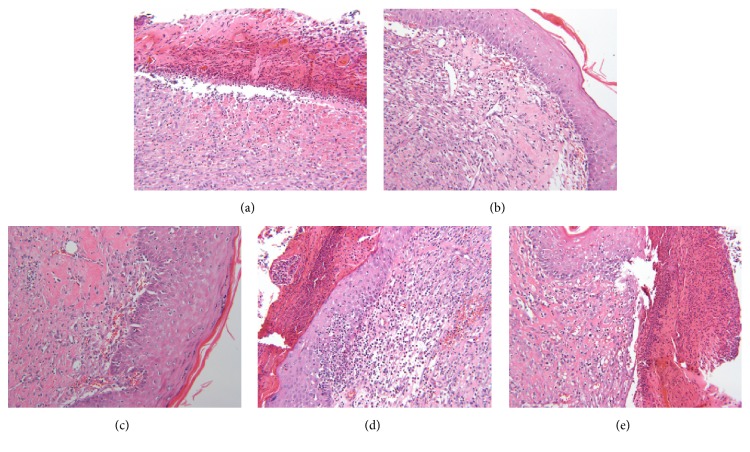
The micrograph of wound tissue sections on day 21. Magnification: × 200. Each picture is represented separately as follows: (a) CON group, (b) 20% CNE group, (c) 10% CNE group, (d) SSD group, (e) Vaseline group.

On the histopathological examination, CON group presented a large area of ulcers involving in inflammatory cells, mild inflammations, moderate dermal bleedings, and severe exfoliation and scabbing, which indicated wound healings were not completed (Figure 3(a)). However, the CNE-treated group demonstrated mildly hypertrophic epidermis and intact dermis obviously distinguished from CON group (Figures 3(b) and 3(c)), which means basic completion of healing. The tissue samples from other groups demonstrated similar results, severe exfoliation, scabbing and bleeding, mild inflammatory cell infiltration, and proliferation of fibrous tissues (Figures 3(d) and 3(e)), which were slightly better than CON group, but the healing was not completed. These results coincided well with gross examination.

### 3.4. Quantitative Analysis of TNF-*α* and IL-10

**Figure 4:**
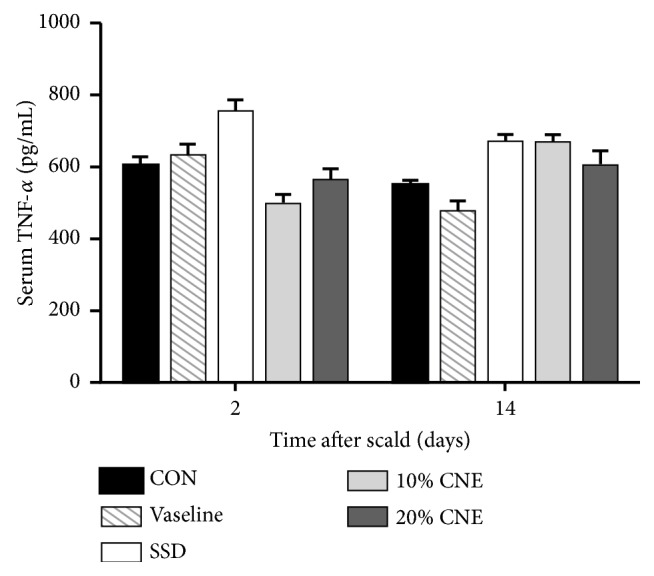
The changes of TNF-*α* level in serum in different groups. CNE: the water extract of* Callicarpa nudiflora*; SSD: silver sulfadiazine; CON: control. Values were represented as the means ± SEM in sextuplicate. *∗p *< 0.05 compared with control.

**Figure 5:**
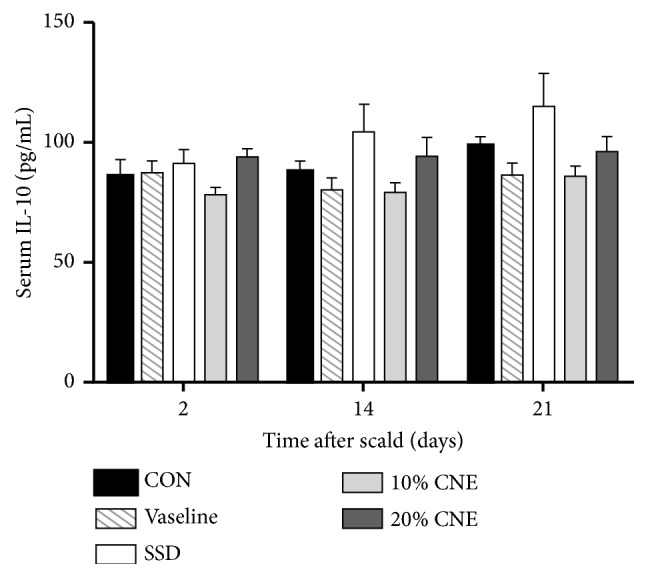
The changes of IL-10 levels in serum in different groups. CNE: the water extract of* Callicarpa nudiflora*; SSD: silver sulfadiazine; CON: control. Values were represented as the means ± SEM in sextuplicate. *∗p *< 0.05 compared with control.

Proinflammatory cytokines have long been deemed to be important factors at wounds involving different reactive processes containing stimulating and regulating of the immune responses, synthesizing and breaking down extracellular matrix proteins, and fibroblast chemotaxis [22]. TNF-*α* of the proinflammatory cytokine was showed to initiate early wound healing responses by upregulating inflammatory phases [23, 24]. Anti-inflammatory cytokines also play an important role in wound repair in addition to proinflammatory cytokines. Especially IL-10 was studied in some detail for wound healing responses [22]. Its primary effect was seemingly due to limitation and eventual termination of inflammatory response. It was reported that IL-10 could lead to reduce matrix deposition and heal the scar [25, 26]. In this experiment, serum was collected on the second and 14th days to explore active effects of TNF-*α* and IL-10 on the inflammatory response. On the 2nd and 14th days after scalding, the levels of TNF-*α* in all groups were at the same level without significant difference (Figure 4). Moreover, there also were no significant difference in serum level of IL-10, the same as that of TNF-*α* shown in Figure 5. These results indicated that CNE might have no effect on regulating the serum level of TNF-*α* and IL-10. CNE seemed to exert wound treatment effect through other signaling pathways.

### 3.5. Quantitative Analysis of TGF-*β*1

**Figure 6:**
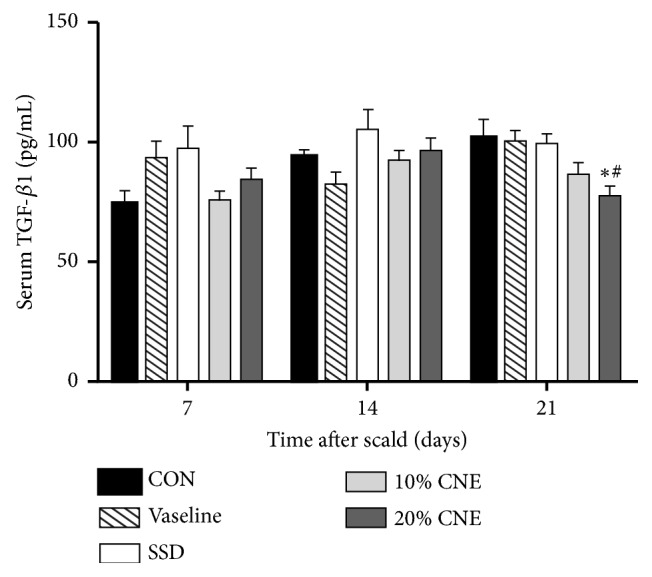
The changes of TGF-*β*1 levels in serum in different groups. CNE: the water extract of* Callicarpa nudiflora*; SSD: silver sulfadiazine; CON: control. Values were represented as the means ± SEM in sextuplicate. *∗p *< 0.05 compared with control and ^#^*p *< 0.05 compared with Vaseline.

The TGF-*β* superfamily encompasses plenty of active factors with diverse activities. These factors appear to be involved in many processes of tissue development and repair [27]. TGF-*β*1 as the key factor in TGF-*β* superfamily plays an important role in healing wound actions containing collagen synthesis, angiogenesis, extracellular matrix deposition, and scar formation [28]. However, if TGF-*β*1 is overexpressed it may result in overhealing [29]. TGF-*β*1 had been shown to participate in scar formation after skin injury in the adult rodents. In the early stage of wound healing, anti-TGF-*β*1 could reduce the scar formation because of reducing inflammatory response and extracellular matrix sediment [30]. Thus, the decreased TGF-*β*1 after reepithelialization might benefit to diminish the appearance of scars. In this experiment, TGF-*β*1 was slightly enhanced in 5 groups except Vaseline-treated group, but only in CNE-treated and SSD-treated groups, the levels decreased after 14 to 21 days. Especially on 21st day, TGF-*β*1 was significantly lower than CON and Vaseline groups in the 20% CNE group (Figure 6). These data could support the results of gross examination that the scabs almost completely disappeared in the CNE groups, but wounds were still evident in CON group (Figure 1).

### 3.6. Quantitative Analysis of VEGF

**Figure 7:**
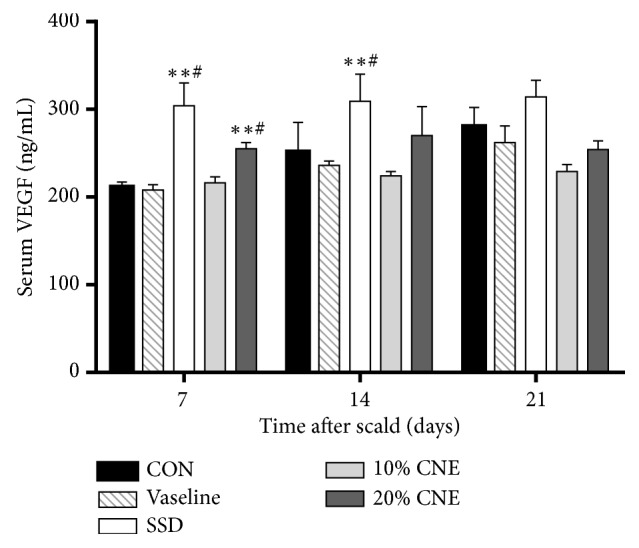
The changes of VEGF levels in serum in different groups. CNE: the water extract of* Callicarpa nudiflora*; SSD: silver sulfadiazine; CON: control. Values were represented as the means ± SEM in sextuplicate. *∗p *< 0.05 and *∗∗p *< 0.01 compared with control. ^#^*p *< 0.05 compared with Vaseline.

Angiogenesis is an important aspect in wound repair. VEGF as a potent proangiogenic growth factor could influence speed and quality of skin repair [31]. VEGF is in favour of wound closure and inadequate VEGF level could cause abnormal wound healing, whereas high VEGF level would exacerbate and induce skin scar formation [32, 33]. In the present study, only on the 7th day 20% CNE-treated group exhibited higher level of VEGF than CON and Vaseline groups with significant difference. The VEGF levels of all groups had no differences on 14th and 21st days (Figure 7). These results suggested that CNE could accelerate the healing of the wound by regulating VEGF in the early stage of healing process.

### 3.7. Smad3 Expression in Level

**Figure 8:**
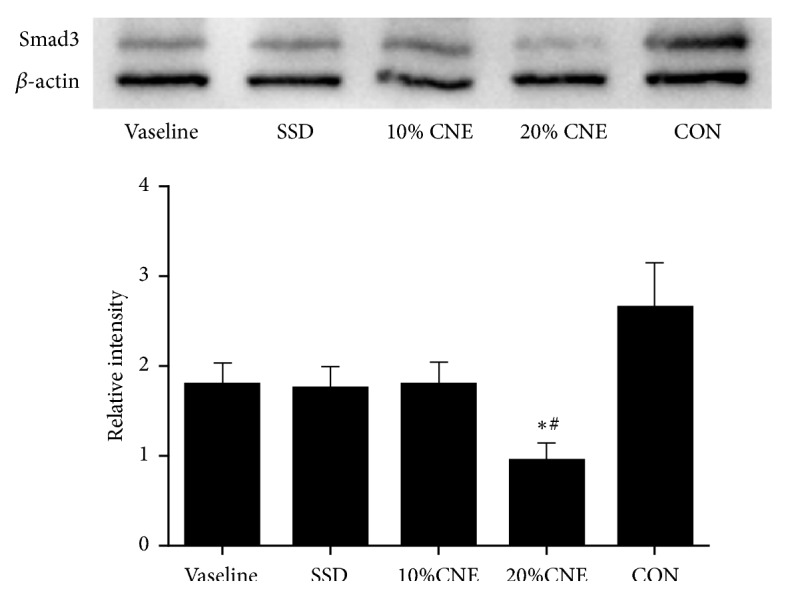
Smad3 protein expressions in different groups. CON, control; SSD, silver sulfadiazine; CNE, the water extract of* Callicarpa nudiflora*. Total proteins (45 *μ*g) were isolated by SDS-PAGE and electroblotted to polyvinylidene difluoride membrane. Smad3 protein levels were defined as the relative values of the *β*-actin protein. Data were represented as the means ± SEM. *∗p *< 0.05. And ^#^*p *< 0.05 compared with Vaseline.

In normal skin fibroblast, Smad3 function is the pivotal endocellular signal sensor to modulate profibrotic TGF-*β* response. TGF-*β*-Smad3 signaling activation seems to be complex epithelial-mesenchymal interactions. The key fibrosis parameters in the dermis containing collagen and myofibroblast accumulation were significantly decreased in the absence of Smad3. These findings suggested the suppression of TGF-*β*-Smad3 signal could be used as a novel approach in keloid therapy [34, 35]. Smad3 expression in scar formation was determined to study influences of CNE on TGF-*β*-Smad3 signal on day 21. As shown in Figure 8, the Smad3 expression was suppressed in different degrees in ointment groups compared to CON group. Furthermore, Samd3 expression level was strongly inhibited by 20% CNE compared to Vaseline and the test showed significant difference between 20% CNE-treated group and CON group in expression levels of Smad3. *β*-actin as an internal control was also determined in the same blot. The result suggested CNE could exert inhibitory effect on expression of Smad3 through TGF-*β*-Smad3 signaling pathway.

### 3.8. Preliminary Phytochemical Screening

The water extract of* Callicarpa nudiflora* was usually used for some qualitative and quantitative phytochemical researches [36–38]. The preliminary phytochemical screening indicated that* Callicarpa nudiflora* contained acteoside, isoacteoside, luteoloside, samioside, rhamnazin, isorhamnetin, philonotisflavone, and other flavonoids and triterpenoids [39, 40]. Acteoside as main constituent of CNE had been confirmed in vitro and in vivo anti-inflammatory, wound healing, and hemostasis [16, 17, 40–42]. Simultaneously, luteoloside is also known anti-inflammatory compound [18]. Therefore, acteoside and luteoloside could be the beneficial components in CNE. Some researches expounded that phytochemical compounds such as triterpenoids and flavonoids were of antimicrobial and astringent effects thereby promoting wound contraction and epithelialization [43, 44]. So other known and unknown constituents in CNE also seem likely to be of wound healing effects as well.

## 4. Conclusions

The results of current experiments indicated that wound healing effects of CNE were quicker and better than Vaseline and SSD in a deep second-degree scald rat model. Macroscopic difference of wound closure in proliferation and tissue remodeling phase suggested a positive effect of CNE on wound closure after 12 days since scald wound induction. The 20% CNE-treated group exhibited structures of epidermis and dermis were well restored with minimum of scars on the last phase of healing. These effects might be due to the upregulation of VEGF on the early stage. In addition, downregulation of TGF-*β*1 and Smad3 expressions might be the main reason for scar reduction by means of CNE treatment.

## Data Availability

The data used to support the findings of this study are available from the corresponding author upon request.

## References

[1] Xu H.-L., Chen P.-P., Wang L.-F. (2018). Skin-permeable liposome improved stability and permeability of bFGF against skin of mice with deep second degree scald to promote hair follicle neogenesis through inhibition of scar formation. *Colloids and Surfaces B: Biointerfaces*.

[2] Lee Jin-Ho, Lee Kyungjin, Lee Mi-Hwa (2017). Wound healing effects of* Prunus yedoensis* matsumura bark in scalded rats. *Evidence-Based Complementary and Alternative Medicine*.

[3] Riedel K., Ryssel H., Koellensperger E., Germann G., Kremer T. (2008). Pathogenesis of chronic wounds. *Der Chirurg*.

[4] Huang S.-H., Lin C.-H., Chang K.-P. (2014). Clinical evaluation comparing the efficacy of Aquacel Ag with Vaseline gauze versus 1% silver sulfadiazine cream in toxic epidermal necrolysis. *Advances in Skin & Wound Care*.

[5] Fuller F. W. (2009). The side effects of silver sulfadiazine. *Journal of Burn Care & Research*.

[6] Agarwal S., Gawkrodger D. J. (2002). Occupational allergic contact dermatitis to silver and colophonium in a jeweler. *American Journal of Contact Dermatitis*.

[7] Lee A.-R. C., Leem H., Lee J., Park K. C. (2005). Reversal of silver sulfadiazine-impaired wound healing by epidermal growth factor. *Biomaterials*.

[8] Olugbuyiro J. A. O., Abo K. A., Leigh O. O. (2010). Wound healing effect of *Flabellaria paniculata* leaf extracts. *Journal of Ethnopharmacology*.

[9] Silambujanaki P., Bala Tejo Chandra C. H., Anil Kumar K., Chitra V. (2011). Wound healing activity of *Glycosmis arborea* leaf extract in rats. *Journal of Ethnopharmacology*.

[10] Albertyn R., Berg A., Numanoglu A., Rode H. (2015). Traditional burn care in sub-Saharan Africa: a long history with wide acceptance. *Burns*.

[11] Yu X., Guo L., Liu M. (2018). Callicarpa nudiflora loaded on chitosan-collagen/organomontmorillonite composite membrane for antibacterial activity of wound dressing. *International Journal of Biological Macromolecules*.

[12] Lai W. Y., Yang W. L., Liu M. S. (2011). Detection of heavy metal contents in four Li nationality medicine. *China Tropical Medicine*.

[13] Wang Y.-J., Yang Y.-F., Gao D. (2008). Advances in studies on chemical constituents in plants of Callicarpa Linn. and their bioactivities. *Chinese Traditional and Herbal Drugs*.

[14] Yang Z.-M., Huang S., Yan X. J. (2015). Study on coagulation activity of Callicarpa nudiflora. *Agricultural Science & Technology*.

[15] Clark R. A. F. (1985). Cutaneous tissue repair: basic biologic considerations. I. *Journal of the American Academy of Dermatology*.

[16] Akdemir Z., Kahraman Ç., Tatli I. I., Küpeli Akkol E., Süntar I., Keles H. (2011). Bioassay-guided isolation of anti-inflammatory, antinociceptive and wound healer glycosides from the flowers of Verbascum mucronatum Lam.. *Journal of Ethnopharmacology*.

[17] Kim K. H., Kim S., Jung M. Y., Ham I. H., Whang W. K. (2009). Anti-inflammatory phenylpropanoid glycosides from Clerodendron trichotomum leaves. *Archives of Pharmacal Research*.

[18] Lee A. Y., Lee S., Kim H. Y., Lee S., Cho E. J. (2016). Anti-inflammatory effects of luteolin and luteoloside from Taraxacum coreanum in RAW264.7 macrophage cells. *Applied Biological Chemistry*.

[19] Lee K., Lee B., Lee M.-H. (2015). Effect of Ampelopsis Radix on wound healing in scalded rats. *BMC Complementary and Alternative Medicine*.

[20] Frank S., Kämpfer H. (2003). Excisional wound healing: an experimental approach. *Wound Healing: Methods and Protocols*.

[21] Galiano R. D., Michaels V J., Dobryansky M., Levine J. P., Gurtner G. C. (2004). Quantitative and reproducible murine model of excisional wound healing. *Wound Repair and Regeneration*.

[22] Werner S., Grose R. (2003). Regulation of wound healing by growth factors and cytokines. *Physiological Reviews*.

[23] Grellner W., Georg T., Wilske J. (2000). Quantitative analysis of proinflammatory cytokines (IL-1*β*, IL-6, TNF-*α*) in human skin wounds. *Forensic Science International*.

[24] Hübner G., Brauchle M., Smola H., Madlener M., Fässler R., Werner S. (1996). Differential regulation of pro-inflammatory cytokines during wound healing in normal and glucocorticoid-treated mice. *Cytokine*.

[25] Liechty K. W., Kim H. B., Adzick N. S., Crombleholme T. M. (2000). Fetal wound repair results in scar formation in interleukin-10-deficient mice in a syngeneic murine model of scarless fetal wound repair. *Journal of Pediatric Surgery*.

[26] Sato Y., Ohshima T., Kondo T. (1999). Regulatory role of endogenous interleukin-10 in cutaneous inflammatory response of murine wound healing. *Biochemical and Biophysical Research Communications*.

[27] Massagué J. (1990). The transforming growth factor-beta family. *Annual Review of Cell and Developmental Biology*.

[28] Choi B.-M., Kwak H.-J., Jun C.-D. (1996). Control of scarring in adult wounds using antisense transforming growth factor-*β*1 oligodeoxynucleotides. *Immunology & Cell Biology*.

[29] Pakyari M., Farrokhi A., Maharlooei M. K., Ghahary A. (2013). Critical role of transforming growth factor beta in different phases of wound healing. *Advances in Wound Care*.

[30] Shah M., Foreman D. M., Ferguson M. W. J. (1995). Neutralisation of TGF-beta 1 and TGF-beta 2 or exogenous addition of TGF-beta 3 to cutaneous rat wounds reduces scarring. *Journal of Cell Science*.

[31] Johnson K. E., Wilgus T. A. (2014). Vascular endothelial growth factor and angiogenesis in the regulation of cutaneous wound repair. *Advances in Wound Care*.

[32] Gira A. K., Brown L. F., Washington C. V., Cohen C., Arbiser J. L. (2004). Keloids demonstrate high-level epidermal expression of vascular endothelial growth factor. *Journal of the American Academy of Dermatology*.

[33] Salem A., Assaf M., Helmy A. (2009). Role of vascular endothelial growth factor in keloids: a clinicopathologic study. *International Journal of Dermatology*.

[34] Lakos G., Takagawa S., Chen S.-J. (2004). Targeted disruption of TGF-beta/Smad3 signaling modulates skin fibrosis in a mouse model of scleroderma. *The American Journal of Pathology*.

[35] Phan T. T., Lim I. J., Aalami O. (2005). Smad3 signalling plays an important role in keloid pathogenesis via epithelial-mesenchymal interactions. *The Journal of Pathology*.

[36] Dong L., Zhang L., Zhang X., Liu M., Wang J., Wang Y. (2014). Two new 3,4-seco-labdane diterpenoids from Callicarpa nudiflora and their inhibitory activities against nitric oxide production. *Phytochemistry Letters*.

[37] Zhang L., Dong L., Huang J. (2013). 3, 4-seco-Labdane diterpenoids from the leaves of Callicarpa nudiflora and their inhibitory effects on nitric oxide production. *Fitoterapia*.

[38] Zhou Z., Wei X., Fu H., Luo Y. (2013). Chemical constituents of Callicarpa nudiflora and their anti-platelet aggregation activity. *Fitoterapia*.

[39] Luo Y., Ma S., Hu S. (2015). Chemical constituents from Callicarpa nudiflora. *Journal of Chinese medicinal materials*.

[40] Zhang J., Li B., Feng F., Tang Y., Liu W. (2010). Chemical constituents from Callicarpa nudiflora and their hemostatic acitivity. *Zhongguo Zhongyao Zazhi*.

[41] Korkina L. G., Mikhal'chik E. V., Suprun M. V., Pastore S., Dal Toso R. (2007). Molecular mechanisms underlying wound healing and anti-inflammatory properties of naturally occurring biotechnologically produced phenylpropanoid glycosides. *Cellular and Molecular Biology*.

[42] Speranza L., Franceschelli S., Pesce M. (2010). Antiinflammatory effects in THP-1 cells treated with verbascoside. *Phytotherapy Research*.

[43] Scortichini M., Rossi M. P. (1991). Preliminary *in vitro* evaluation of the antimicrobial activity of terpenes and terpenoids towards *Erwinia amylovora* (Burrill) Winslow et al. *Journal of Applied Bacteriology*.

[44] Tsuchiya H., Sato M., Miyazaki T. (1996). Comparative study on the antibacterial activity of phytochemical flavanones against methicillin-resistant *Staphylococcus aureus*. *Journal of Ethnopharmacology*.

